# Near-infrared (NIR) spectroscopy. A new method for arthroscopic evaluation of low grade degenerated cartilage lesions. Results of a pilot study

**DOI:** 10.1186/1471-2474-8-47

**Published:** 2007-05-29

**Authors:** Gunter Spahn, Holger Plettenberg, Enrico Kahl, Hans M Klinger, Thomas Mückley, Gunther O Hofmann

**Affiliations:** 1 Center of Traumatology and Orthopaedic Surgery, Sophienstr. 16, 99817 Eisenach, Germany; 2 Research Center of Medical Technology and Biotechnology, Geranienweg 7, 99947 Bad Langensalza, Germany; 3 Orthopaedic Clinic, Georg August University Göttingen, Postbox 3742, 37070 Göttingen, Germany; 4 Department of Trauma, Friedrich Schiller University of Jena, Erlanger Allee 101, 07747 Jena, Germany; 5 Trauma Center Bergmannstrost Halle, Merseburger Str. 165, 06112 Halle, Germany

## Abstract

**Background:**

Arthroscopy is a highly sensitive method of evaluating high-grade cartilage lesions but the detection of low-grade lesions is often is unreliable. Objective measurements are required. A novel NIRS (near-infrared-spectroscopy) device for detection of low-grade cartilage defects was evaluated in a preliminary clinical study.

**Methods:**

In 12 patients who had undergone arthroscopy, the cartilage lesions within the medial knee compartment were classified according to the ICRS protocol.

With a NIR spectrometer system and an optical probe, similar in design to a hook used for routine arthroscopy, the optical properties of cartilage were measured during arthroscopy.

**Results:**

The mean ratio of 2 NIR absorption bands of intact cartilage 3.8 (range 2.3 to 8.7).was significantly lower than that of cartilage with grade 1 lesions (12.8, range 4.8 to 19.6) and grade 2 lesions (13.4, range 10.4 to 15.4).

No differences were observed between grade 1 and grade 2 lesions.

**Conclusion:**

NIRS can be used to distinguish between ICRS grade 1 lesions and healthy cartilage during arthroscopic surgeries. The results of this clinical study demonstrate the potential of NIRS to objectify classical arthroscopic grading systems.

## Background

Cartilage degeneration is associated with complex changes in cartilage matrix composition that result in decreased collagen and proteoglycan content and increased water content. These changes in matrix composition correlate with decreased mechanical stiffness. As a result, four stages of cartilage lesions develop. The consensus conference of the ICRS I (international Cartilage Repair Society) suggested a score for cartilage lesions in 2002 [1]: The grades were as follows: ICRS grade 0 (normal), ICRS grade 1 (nearly normal, superficial lesions with soft indentation and/or superficial fissures and cracks), ICRS grade 2 (abnormal, lesions extending down to <50% of cartilage depth), ICRS grade 3 (severely abnormal, cartilage defects extending down > 50% of cartilage depth) and ICRS grade 4 (severely abnormal, complete defect).

Clinical examination (crepitus, effusion and pain) is nonspecific. Radiological signs of osteoarthritis (joint space narrowing, subchondral sclerosis, osteophytes) are primarily associated with high-grade cartilage damage, and are therefore inadequate for diagnosing early disease stages.

The diagnosis of cartilage lesions can be made by MRI or arthroscopic evaluation. MRI is the most important non-invasive diagnostic method for assessing cartilage lesions. Many studies have demonstrated the high validity of MRI for evaluating chondral lesions in the knee as well as in other joints, but MRI has low accuracy for initial lesions. Only specific techniques like dGEMRIC (delayed Gadolinium Enhanced MRI of Cartilage) are sufficient for evaluating initial chondral lesions [2-4].

The arthroscopic diagnosis of chondral lesions depends on the surgeon's subjective rating alone. Diagnosis is made by visualizing the lesion on the video monitor and by probing with the arthroscopy hook.

Friemert et al. [5] found a good correlation between MRI and arthroscopic findings in the case of deep cartilage lesions. One criterion for the validity of arthroscopic diagnosis is the "inter-observer-validity". The diagnosis of deep cartilage defects has high validity but the diagnosis of low grade lesions is often inaccurate [6-11]. Detection of these early stages primarily involves palpation with the arthroscopic hook. This palpation depends on the power applied by the manual pressure of the surgeon and, as Li and Herzog demonstrated, on the geometry of the distal end of the hook [12].

Recent studies have used mechanical devices to attempt to measure cartilage softening objectively by evaluating the reduced stiffness of the cartilage [13-17]. Duda et al. created an instrument for stiffness measurements in a "low-contact-modus". The reduction of cartilage stiffness was measured as a function of surface deformation produced by a pulsed flow [18]. However, the practical use of these instruments is limited by their dimensions. Furthermore, the necessity of positioning these instruments exactly vertical to the cartilage surface may limit their practical use in routine arthroscopy.

Early disease stages are characterized by a rise in the water content of the extracellular matrix and, as a result, cartilaginous swelling [19-23]. The water inflow strongly correlates with the loss of mechanical stiffness [24]. In the last decade NIRS (near-infrared-spectroscopy) became an important method for analyzing materials with complex mixes of chemical substances [25], in particular for measuring the water content of the material In a recent ex vivo study Spahn et al. [26] demonstrated a decrease of NIR-absorption in low-grade degenerated cartilage. The decreased NIR absorption did correlate with an increase of water content in early degenerated cartilage.

This study introduces a novel NIRS-device that is capable of identifying low-grade cartilage lesions under arthroscopic control.

## Methods

### Patients

All patients gave informed consent and agreed to participate in the study. This study was approved by the regional Ethics Committee (Jena, RZ 714-0110).

Twelve patients were selected who had been suffering from knee pain for more than 3 months and had undergone arthroscopic evaluation. There were 7 male and 5 female patients. The patients were 31.1 ± 6.7 years old (range 25 to 45). No patient had suffered from an injury. No patient had undergone other prior surgery. All patients had a non-traumatic medial meniscus tear and had undergone partial meniscectomy during arthroscopy.

### Preoperative Imaging

The preoperative diagnosis was made by clinical examination, radiography and MRI. All patients had suffered from knee pain and demonstrated clinical signs of medial meniscus tears. No patient had radiological signs of osteoarthritis on standard x-ray [27].

The cartilage defects were graded by MRI according to Vallotton et al. [28]. No patient had abnormal MRI findings in the lateral compartment or within the femoropatellar joint. The evaluations of the radiographs and MRI scans were performed by author EK.

### Arthroscopic evaluation

All operations were performed under general anesthesia by using a tourniquet. The tourniquet was placed halfway between the knee and hip. A standard 0.9 % sodium chloride solution was used for irrigation. A lavage (p = 80 mmHg, flow = 100 ml/min) was performed for exactly 3 minutes before NIR measurements to remove any joint effusion.

The arthroscopic evaluation began with visualization of all joint compartments and included palpation with an arthroscopic hook. The cartilage defects were graded according to the ICRS protocol [1] by visualization and probing with an arthroscopic hook : ICRS grade 0 (normal), ICRS grade 1 (nearly normal, superficial lesions with soft indentation and/or superficial fissures and cracks), ICRS grade 2 (abnormal, lesions extending down to <50% of cartilage depth.

The ICRS grade classifications were determined independently by author MK, whereas the NIR measurements were performed independently by GS. Both are experienced arthroscopic surgeons who have performed more than 10.000 knee arthroscopies.

### NIR spectroscopic evaluation

**Figure 1:**
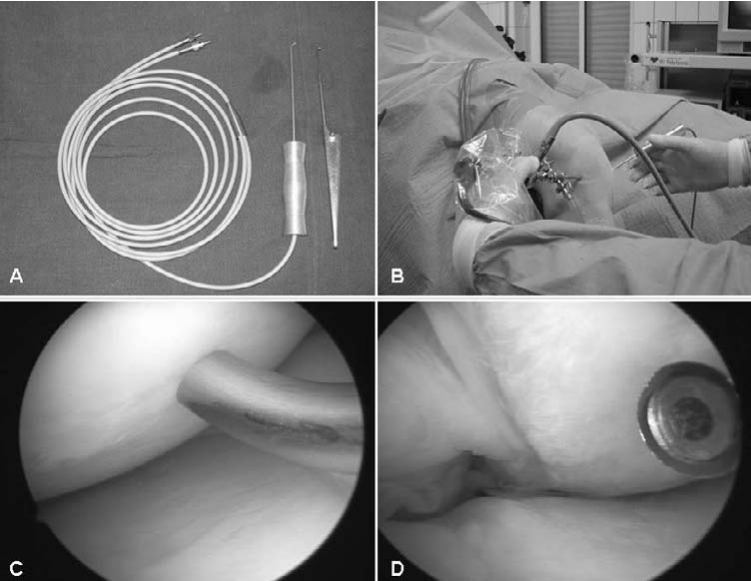
**NIRS for evaluation of cartilage lesions**. Light from a stabilized light source was coupled into six optical fibers. The collection fiber (silica glass, 200 μm diameter) was connected to the spectrometer. The fibers were combined in a reflection-probe with the light delivering fibers surrounding the collection fiber. The design of the probe was similar to a routine arthroscopic hook (A). The intraoperative measurements were performed by using the probe in the same way as a hook (B and C). The top of the hook shows the end of the optical fibers (D).

After routine arthroscopic evaluation the medial femoral condyle and the medial tibial surface within the medial bearing zone.and within the non-weight-bearing margin were examined with a NIR probe (Figure 1).

The main components of the NIR spectrometer system were a diode micro-spectrometer (microparts, Dortmund, Germany) with a spectral range of 1100 nm to 1700 nm and spectral resolution of approx 16 nm, a stabilized light source (LQ2NIR, JETI Technische Instrumente GmbH, Jena, Germany) and a fibre optical reflection probe (Loptec, Berlin, Germany) with six fibers (silica glass, 200 μm diameters) for illumination surrounding one collection fibre (silica glass, 200 μm diameter)[26]. The probe's design is similar to that of a hook used for routine arthroscopy. The whole NIR spectrometer system (optical and electrical parts) is in accordance with the German law for medical products (Medical Device Directive (MDD) 93/42/EEC) [29].

Prior to each NIR measurement a reference spectrum was recorded in the irrigation solution within the superior recessus. Then the tip of the probe was placed on the cartilage surface in the region of interest and 10 NIR reflection spectra (cartilage spectrum) per second were recorded continuously. A total of 50 spectra were taken per region and averaged. Absorption spectra were then calculated [absorption spectrum = -log_10 _(cartilage spectrum/reference spectrum). The ratio (R) of the peak absorptions of two bands (the 1^st ^OH and CH combination overtones (1340 nm – 1475 nm) and the 2^nd ^CH overtone (1150 nm – 1220 nm) was calculated for statistical evaluation. This ratio (absorption at 1425 nm/absorption at 1175 nm) represents the relative proportion of water to organic substances and can therefore be regarded as an indicator of the water content within the cartilage [29]. Evaluation of NIR measurements was completed independently by author HP.

### Statistical analysis

The results of every diagnostic tool were blinded before final evaluation.

Statistical analyses were performed on a personal computer using SPSS (13.0), SPSS Inc., Chicago Illinois. After testing for normal distribution and variance homogeneity, a One-Way Analysis of Variances (ANOVA) and post – hoc pairwise comparison of means were preformed. The Pearson correlation coefficients were used to examine the relationships between the parameters. A p value < 0.05 was considered significant.

## Results

### Radiography and MRI evaluation

No patient showed any signs of osteoarthritis in standing radiographs. The width of the joint space did not correlate with intra-articular findings or with the BMI (table 1 and 2).

In routine MRI ICRS grade 0 cartilage was correctly classified 15 times, but grade 0 lesions were overestimated 13 times. Grade 1 lesions were correctly judged 13 times and underestimated 1 time. Of the grade 2 lesions, two defects were correctly classified but 3 were underestimated (table 2).

There was no correlation of BMI with the width of the joint space in standing radiographs (R = -0.232, p = 0.469). The width of the joint space also did not correlate with the grade of cartilage lesions (R = 0.225, p = 0.482).

### Arthroscopic findings

**Figure 2:**
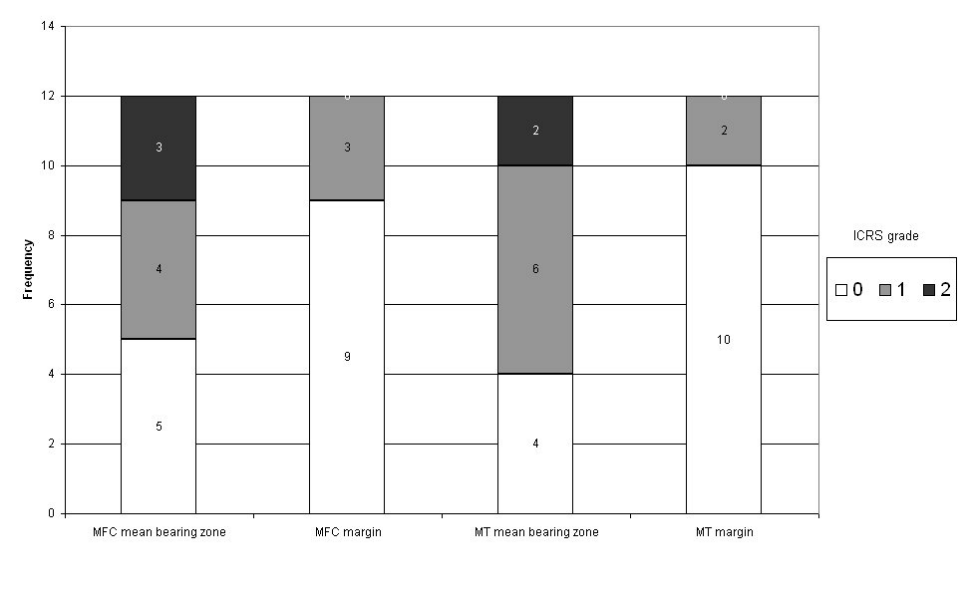
Distribution of cartilage lesions.

A serious effusion (more than 10 ml) was detected in 4 patients and 3 patients had a mild synovialitis. The distribution of cartilage lesions within the medial joint compartment is shown in figure 2. Cartilage lesions within the mean weight-bearing zone were significantly higher graded than in the margin (p = 0.027). No patient had cartilage lesions within the patello-femoral or lateral joint compartment. All patients had suffered a medial meniscus tear that required partial meniscectomy (table 1).

### Reference spectra in NIRS

The mean reference spectra recorded from saline irrigation solution within the superior recessus totaled 124.7 ± 27.8 Counts. No differences were identified between this control and joint effusions (p = 0.298) or synovialitis (p = 0.166).

### Evaluation of low grade cartilage lesions by NIR spectroscopy

The ratio (R) of NIR absorption (AU@1425 nm/AU@1175 nm) within intact cartilage was 3.8 ± 1.2 (range 2.3 to 8.7). In cartilage lesions it was significantly higher than in intact cartilage, (p = 0.000, table 3) with values for grade 1 lesions of 12.8 ± 4.8 (range 4.8 to 19.6) and for grade 2 lesions of 13.4 ± 2.1 (range 10.4 to 15.4).

No differences in R were observed between grade 1 and grade 2 lesions (p = 0.828).

**Figure 3:**
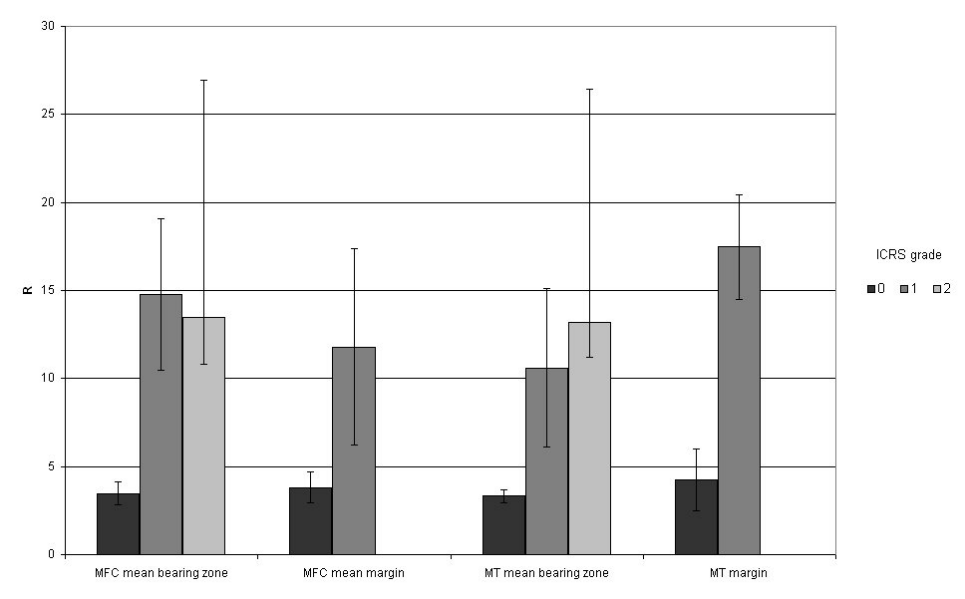
**Results of NIRS probing within the cartilage surfaces of the medial joint compartment**. The ratio of NIR absorption (R) within grade 1 lesions was significantly higher than in intact cartilage.

R depended on the grade of cartilage lesions but not on the area of measurement (p = 0.244), figure 3.

There were no differences in R between male and female patients (p = 0.057) and no correlations to the patient's age (p = 0.282).

### Validity of MRI and NIRS in evaluation of initial cartilage lesions

The cartilage lesions in a total of 48 regions were evaluated by MRI, arthroscopy and NIRS. The arthroscopic diagnosis made by a "highly experienced" arthroscopic surgeon was defined as the diagnostic standard for the cartilage lesions.

In routine MRI the ICRS grade 0 cartilages were classified correctly 15 times, but grade 0 lesions were overestimated 13 times. Grade 1 lesions were correctly judged 13 times and underestimated 1 time. Of the grade 2 lesions, 2 defects were correctly classified but were underestimated 3 times.

The "normal-value [mean ± 2 SD]" of R for intact cartilage was 1.4 to 6.2. The 27 normal cartilages (grade 0) had values of R within the normal value excepting one outlier with a higher R. The values of R were always higher for cartilage lesions than for intact cartilage. NIRS has a significantly (p = 0.000) higher accuracy (0.979) than MRI (0.708). The diagnostic parameters of MRI and NIRS are listed in table 4.

Though NIRS and MRI show a similar sensitivity (1 and 0.95), NIRS has a significantly (p = 0.000) higher specificity (0.96) and accuracy (0,98) than MRI (0.54 and 0.70 respectively).

## Discussion

This study reports the possibility of using NIRS for objective identification of low-grade cartilage lesions during routine arthroscopy. We hypothesized that complex changes in cartilage matrix composition are reflected by changes in chondral optical properties in the NIR region.

The diagnosis of cartilage lesions can be made by MRI as well as arthroscopic evaluation. MRI using "routine sequences" has good validity for diagnosis of deep cartilage lesions. In contrast it is relatively inaccurate for evaluation of low grade lesions or validation of intact cartilage [28-36].

Our results confirm this. Our MRI scans had excellent sensitivity for low grade cartilage lesions (0.950) but poor specificity (0.536).

In the future, special MRI techniques for cartilage lesions like d-Gemric (delayed Gadolinium Enhanced MRI of Cartilage) may improve the quality of these evaluations.

The arthroscopic evaluation of cartilage lesions is the current "gold standard". In standard arthroscopy cartilage lesions are graded using semiquantitative scores (eg Outerbridge classifications, ICRS score or others) [1,37,38]. The validity of this evaluation depends on the surgeon's experience. High-grade lesions are relatively obvious but the diagnosis of low-grade lesions is often difficult. Thus, other investigators have used mechanical stiffness measurements for evaluating softening in low-grade cartilage degeneration. However, the necessity of placing the stiffness probe exactly vertical to the chondral surface is disadvantageous [39] and can introduce difficulty during arthroscopic operations.

NIRS offers the possibility of evaluating changes in material composition [25]. Since water has a particularly high NIR absorption, NIRS is able to analyze water content in composite materials.

In the present study probes curved and dimensioned like a normal arthroscopic hook were used to record NIR reflection spectra of cartilage. It was therefore possible to reach all regions of interest without difficulty. The handling of the probe is easy, with measurement times of approx. 5 s per region. Probes can be sterilized like any other arthroscopic instruments. In contrast to histological, pathological, biochemical and mechanical measurements NIRS is a completely non-destructive method.

By using NIRS the surgeon can distinguish between healthy and low-grade cartilage lesions, but not between high-grade lesions. This may be due to the fact that a water inflow is characteristic of the initial phase of degeneration but not of more advanced stages of chondral disease. Furthermore, at higher grades of degeneration the surface of cartilage becomes irregular, disturbing the collection of reflection spectra. Thus NIRS is restricted to detection of lower grade cartilage damage. Future investigations may identify NIR equipment and bands that can be used to characterize cartilage lesions with higher grades, although such lesions are easily detected by visualization even by relatively inexperienced surgeons.

Although the cartilage lesions were graded by a highly-experienced arthroscopic surgeon (MK), these grades are still subjective and therefore stand as the principal point of critique in our study. In contrast, the NIRS evaluations were made independently from the standard cartilage grading. The high concordance between arthroscopic grading and NIRS may favor this technique for future use in arthroscopy.

## Conclusion

Our results suggest that NIRS could become an adequate method for arthroscopic differentiation of healthy and low-grade degenerated cartilage in future. Of course this is a preliminary study in only 12 patients. Because the study was aimed to check the principle of NIR measurements in cartilage evaluation, the clinical relevance of the cartilage lesions wasn't evaluated. In future larger series with independent investigators are required. NIRS measures the complex changes in the composition of low-grade degenerated cartilage. Thus it is possible that this method can be used routinely in arthroscopy in the future.

## Abbreviations

ANOVA Analysis of Variances

d-Gemric delayed Gadolinium Enhanced MRI of Cartilage

ICRS International cartilage repair society

MRI Magnetic resonance imaging

NIR Near infrared

NIRS Near infrared spectroscopy

## Competing interests

The author(s) declare that they have no competing interests.

## Authors' contributions

Dr. med. Gunter Spahn Arthroscopic operations, manuscript

Holger Plettenberg NIR Analysis, technical support

Dr. med. Enrico Kahl Radiological and MRI evaluation

Dr. med. Hans Michael Klinger Independent cartilage evaluation, statistics

Dr. med. Thomas Mückley Manuscript check

Prof. Dr. Dr. Gunther O. Hofmann Manuscript check

All authors read and approved the final manuscript.

## Pre-publication history

The pre-publication history for this paper can be accessed here:



## Figures and Tables

**Table 1:** 

	**Sex**	**Age [years]**	**BMI [kg/m^2^]**	**Arthroscopic findings**		
				
				**Effusion**	**Synovialitis**	**Form of medial meniscus tear**
LE	female	40	26.7			radial
DB	female	41	25.9		+	buckle handle
RK	male	35	28.1			flap
RM	male	28	24.5			horizontal
HW	male	32	29.4	+	+	horizontal
LS	male	27	27.3		+	radial
GE	male	32	29.2			horizontal
SD	male	28	24.4	+		horizontal
MJ	female	25	26.4	+		radial
LC	female	45	23.2	+		flap
SJ	male	26	29.1			flap
MA	female	26	25.7			radial

**Table 2:** Results of preoperative imaging

	**Sex**	**Age [years]**	**Width of medial joint space [mm]**	**MFC mean bearing zone**		**MFC margin**		**MT mean bearing zone**		**MT margin**	
				
				ICRS grade	MRI grade	ICRS grade	MRI grade	ICRS grade	MRI grade	ICRS grade	MRI grade
LE	female	40	7	1	1	0	1	1	1	0	1
DB	female	41	6	0	1	0	1	0	0	0	0
RK	male	35	7	0	0	0	1	0	1	0	1
RM	male	28	8	2	1	0	0	1	1	0	0
HW	male	32	8	2	2	0	0	1	2	0	1
LS	male	27	6	0	0	0	1	0	0	0	1
GE	male	32	7	0	1	0	0	0	1	0	0
SD	male	28	11	1	1	0	0	2	1	0	0
MJ	female	25	9	0	0	0	0	1	0	0	0
LC	female	45	6	1	1	1	1	1	1	1	1
SJ	male	26	7	2	1	1	1	2	2	0	1
MA	female	26	10	1	1	1	1	1	1	1	1

**Table 3:** Results of NIRS for evaluating cartilage lesions

	**Sex**	**Age [years]**	**MFC mean bearing zone**		**MFC margin**		**MT mean bearing zone**		**MT margin**	
			
			ICRS grade	R*	ICRS grade	R**	ICRS grade	R***	ICRS grade	R****
LE	female	40	1	16,7	0	2,3	1	12,7	0	2,3
DB	female	41	0	2,5	0	2,6	0	3,0	0	3,4
RK	male	35	0	3,5	0	3,4	0	3,1	0	3,2
RM	male	28	2	14,6	0	4,0	1	5,5	0	3,1
HW	male	32	2	15,4	0	4,1	0	4,8	0	4,9
LS	male	27	0	4,3	0	4,2	0	3,8	0	4,2
GE	male	32	0	3,5	0	4,4	0	3,4	0	4,2
SD	male	28	1	14,8	0	4,6	2	14,6	0	4,3
MJ	female	25	0	3,6	0	4,8	1	15,1	0	4,2
LC	female	45	1	8,8	1	8,4	1	10,6	1	15,4
SJ	male	26	2	10,4	1	8,8	2	11,8	1	8,7
MA	female	26	1	18,8	1	18,2	1	14,8	1	19,6

p < 0.05*, p < 0.02 **, p < 0.001; ***, p < 0.000****

**Table 4:** Validity of MRI and NIRS in evaluation of low-grade cartilage lesions

	MRI	NIRS
Correctly positive test	19	20
Correctly negative test	15	27
False positive test	13	1
False negative test	1	0
Sensitivity	0.950	1.000
Specificity	0.536	0.964
Positive predictive value	0.678	0.714
Negative predictive value	0.937	1.000
Likelihood ratio positive test	2.05	27.7
Likelihood ratio negative test	0.093	∞
Accuracy	0.708	0.979
